# Oncological and functional outcomes of organ‐preserving cystectomy versus standard radical cystectomy: A systematic review and meta‐analysis

**DOI:** 10.1002/bco2.189

**Published:** 2022-10-03

**Authors:** Reece Clay, Raghav Shaunak, Siddarth Raj, Alexander Light, Sachin Malde, Ramesh Thurairaja, Oussama El‐Hage, Prokar Dasgupta, Muhammed Shamim Khan, Rajesh Nair

**Affiliations:** ^1^ GKT School of Medical Education King's College London London UK; ^2^ William Harvey Hospital East Kent Hospital University Foundation Trust Kent UK; ^3^ St Peter's Hospital Ashford and St Peter's Hospital NHS Trust Chertsey UK; ^4^ University Hospital University Hospitals Coventry and Warwickshire NHS Trust Coventry UK; ^5^ Imperial College London London UK; ^6^ Department of Surgery and Cancer Imperial College Healthcare NHS Trust London UK; ^7^ The Urology Centre, Guy's and St. Thomas' NHS Foundation Trust Guy's Hospital London UK

**Keywords:** continence, cystectomy, functional, oncological, organ sparing, radical, review, sexual, urology

## Abstract

**Introduction:**

Radical cystectomy (RC) is historically considered the gold standard treatment for muscle invasive and high‐risk non‐muscle invasive bladder cancer. However, this technique leaves the majority of patients of both sexes with poor sexual and urinary function. Organ‐sparing cystectomy (OSC) techniques are emerging as an alternative to the standard procedure to preserve these functions, without compromising the oncological outcomes. We present a systematic review and meta‐analysis of the published literature.

**Methods:**

MEDLINE, Embase and Web of Science were systematically searched for eligible studies on 6 April 2021. Primary outcomes studied were both oncological outcomes, specifically overall recurrence, and functional outcomes, specifically sexual function, and daytime and nighttime continence. Odds ratios (OR) with 95% confidence intervals (95% CI) were calculated. The PROSPERO registration reference number was CRD42018118897.

**Results:**

From 13 894 identified abstracts, 19 studies (1886 male and 305 female patients) were eligible for inclusion in this review. These studies included patients who underwent either whole prostate, prostate capsule, seminal vesicle, nerve, uterus, ovary, vagina and fallopian tube sparing techniques. Four studies included only female patients.

Thirteen studies reported oncological outcomes, and overall recurrence rate was similar between the two groups (five studies; OR 0.73; 95% CI 0.38–1.40, *p* = 0.34). Thirteen studies reported on male sexual function. In men, OSC had significantly greater odds of retaining potency (five studies; OR 9.05; 95% CI 5.07–16.16, *p* < 0.00001). Fourteen studies (13 on males and 1 female) reported urinary outcomes. In men, OSC demonstrated greater odds of daytime (seven studies; OR 2.61; 95% CI 1.74 to 3.92, *p* < 0.00001) and nighttime continence (seven studies; OR 2.62; 95% CI 1.76 to 3.89, *p* < 0.00001).

**Conclusion:**

In carefully selected patients, OSC allows the potential to provide better sexual and urinary function without compromising oncological outcomes. There remains, however, a paucity of OSC studies in females. Further studies are required to make recommendations based on robust clinical evidence.

## 1 INTRODUCTION

**FIGURE 1:**
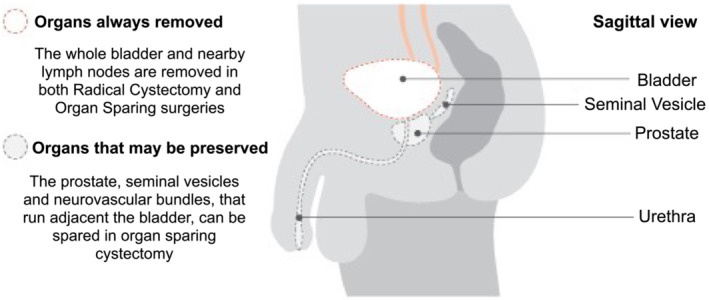
Diagram of male pelvic anatomy^3^

**FIGURE 2:**
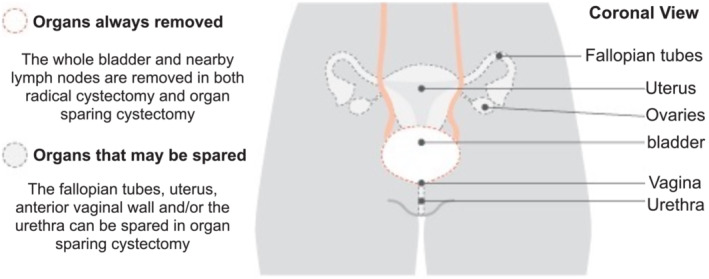
Diagram of female pelvic anatomy^3^

Radical cystectomy is the gold standard surgical treatment for patients with muscle‐invasive bladder cancer, or with non‐muscle invasive bladder cancer but at high risk of progression, or after failure of intravesical Bacillus Calmette‐Guérin (BCG)  therapy.^1^, ^2^ In males, this operation involves removal of the bladder, prostate gland, seminal vesicles and neurovascular bundles (Figure 1). In females, the radical surgery involves removal of the bladder, uterus, ovaries and anterior vaginal wall (Figure 2). However, this *en bloc* removal of the organs results in debilitating sexual dysfunction with serious lifestyle implications in younger patients.^3^, ^4^, ^5^

First described by Marshall and Whitmore in the 1940s, open RC was the primary surgical technique to treat muscle invasive bladder cancer—though it carried significant perioperative morbidity and mortality.^6^ The advent of minimally invasive/laparoscopic cystectomy in the 1990s aimed to reduce adverse outcomes. Recent randomised control trials demonstrated fewer complications whilst achieving comparable oncological outcomes, though requiring longer operating times.^7^ Owing to technological advancements, robotic‐assisted RC has grown in popularity in high volume centres. Numerous studies highlight similar oncological outcomes and lower adverse outcomes, with the potential to improve surgeon precision and dexterity whilst reducing surgeon fatigue, though higher operating costs have restricted its widespread adoption.^8^

Because of increasing awareness and concerns about functional implications of standard RC, there is growing interest in organ‐sparing cystectomy (OSC) techniques. These techniques aim to maintain similar oncological outcomes to RC but with improvement in sexual and urinary outcomes. In males, prostate‐sparing cystectomy was first described in 2002 and involves sparing the prostate gland, seminal vesicles, vas deferens and neurovascular bundles.^9^ Alternative techniques include capsule‐sparing cystectomy, where just the prostatic capsule is preserved after enucleating the inner part of the prostate gland.^10^, ^11^, ^12^ In seminal vesicle‐sparing cystectomy, the seminal vesicles, vas deferens and neurovascular bundles are preserved.^13^, ^14^ Lastly, in the neurovascular bundle‐sparing cystectomy, the nerve bundles are preserved.^15^

In females, organ‐sparing techniques are less well described. These have included sparing of the uterus, fallopian tubes, ovaries and anterior wall of the vagina.^16^ Alternative techniques include vaginal‐sparing cystectomy, where the anterior wall of the vagina is preserved,^17^ and neurovascular bundle‐sparing cystectomy.^18^

Given the relative paucity of organ‐preserving cystectomies, there is a lack of consensus in regard to the benefit of these techniques in the holistic management of bladder cancer patients. The aim of this systematic review is to provide an update of results on the role of organ‐preserving techniques in the management of bladder cancer patients of either sex suitable for OSC.

## 2 MATERIALS AND METHODS

### 2.1 Search strategy

The protocol for this review has been published online in the PROSPERO database (CRD42018118897).^19^ MEDLINE, Embase and Web of Science were systematically searched for eligible studies on 6 April 2021. Only articles from the year 2000 onwards were included in order to reflect modern practice. The article search and selection was based on the Preferred Reporting Items for Systematic Reviews and Meta‐analyses criteria. We also consulted experts, reviewed reference lists from all eligible articles and reviewed the European Association of Urology guidelines on bladder cancer.

**FIGURE 3:**
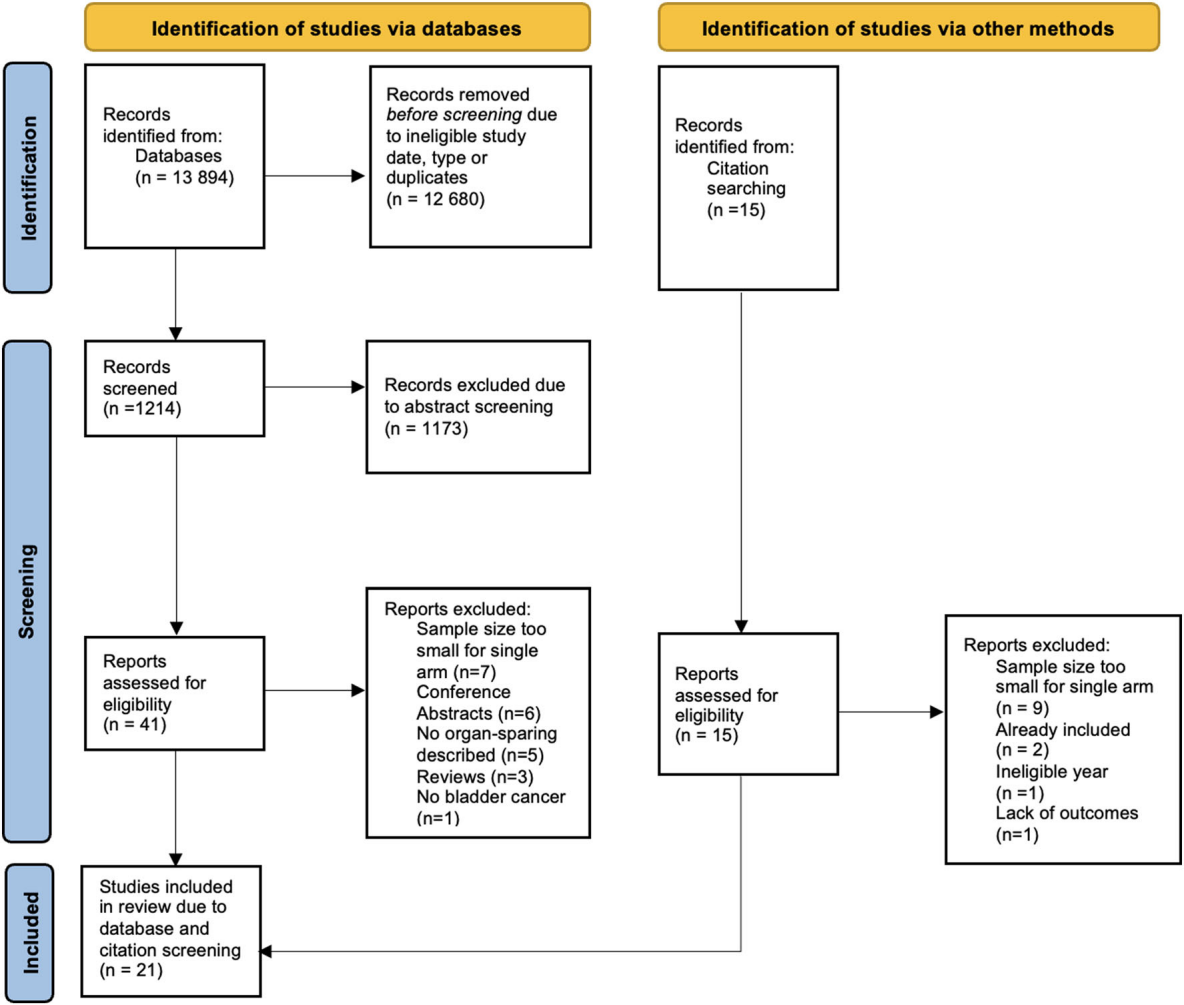
PRISMA flow diagram of studies identified, included and excluded. PRISMA, Preferred Reporting Items for Systematic Reviews and Meta‐analyses

Three reviewers (R.S., R.C. and S.R.) screened the article titles identified from the database search for potentially eligible articles. Once eligible articles had been identified, further abstract screening was commenced with the final list of papers undergoing a full text screen. Those shortlisted had their full manuscript assessed against eligibility criteria (Figure 3). Any disagreement was resolved by a fourth reviewer (A.L.). Data from eligible articles were entered into a piloted, predefined spreadsheet, which was then independently reviewed by the fourth reviewer (A.L.).

### 2.2 Eligible study types

All study designs comparing the relevant interventions were considered for inclusion. Case series involving OSC were included if they involved over 50 patients. Studies reported in English, or any other language with an accessible English translation, involving human participants since 2000 were included.

### 2.3 Eligible participants

The participants of interest were patients, male or female, with bladder cancer undergoing cystectomy for curative intent in the primary setting. The patients with bladder cancer, either muscle‐invasive (MIBC) or high‐risk non‐muscle‐invasive (NMIBC), and any stage up to T4 Nx/N1M0 undergoing cystectomy were included. Adjuvant treatments were permissible.

### 2.4 Eligible interventions and comparators

The intervention of interest was pelvic OSC. For men, this included prostate‐sparing, capsule‐sparing, seminal vesicle‐sparing and neurovascular bundle‐sparing cystectomies. In women, this included uterus‐sparing, ovary‐sparing, vagina‐sparing cystectomy and neurovascular bundle‐sparing cystectomy. The comparator was standard RC, where pelvic organ sparing was not attempted.

We also included studies that compared individual organ‐sparing techniques, without a comparison to standard RC, for example, a study comparing capsule‐sparing cystectomy and seminal vesicle‐sparing cystectomy. As mentioned, we also included case series of a single technique if over 50 patients were included.

### 2.5 Outcome measures

Primary outcomes of interest were both oncological and functional. The oncological outcomes were assessed by reported positive surgical margins, local recurrence or metastatic disease (including site and time of diagnosis), summated overall recurrence, recurrence‐free survival, progression‐free survival disease‐free survival and overall survival after 2 years. The data regarding adjuvant treatments and proportion of orthotopic neobladders were also collected.

The primary functional outcome was sexual function after surgery, as compared to preoperative status. In males, sexual function (potency) was assessed from subjective reporting on erectile function and questionnaires such as International Index of Erectile Function‐5 (IIEF‐5). We also planned to analyse fertility data, ability to achieve orgasm, and adjuvant treatments. For female patients, we extracted data pertaining to vaginal length, ability to have penetrative sex and the use of hormone‐replacement therapy.

The secondary outcomes were measures of urinary function, including day and night continence (as measured by questionnaires), number of pads used and self‐reported outcomes. Urodynamic study data were also extracted in patients who underwent orthotopic neobladder formation or other functional assessments.

### 2.6 Assessment of risk of bias

This was dependent on the study design. The Cochrane Risk of Bias 2.0 tool was used to assess randomised‐controlled trials (RCTs),^20^ the risk of bias in non‐randomised studies of interventions tool for non‐randomised comparative studies (ROBINS‐I), and the Murad tool for single‐arm case series.^21^, ^22^

### 2.7 Subgroup analyses

Because of the nature of the intervention, male and female patients were assessed separately. We also performed subgroup analysis according to the type of OSC, the modality (robot assisted, laparoscopic and open) and according to differences in how functional outcomes were measured, for example, using questionnaires, subjective reports or other measures.

### 2.8 Data analysis

A qualitative synthesis was performed, with discussion of possible explanations, and a subsequent summation in the conclusion. Meta‐analysis was performed to compare local, metastatic, and overall recurrence rates; potency; and day and night continence at 12 months. Odds radios (OR) with 95% confidence intervals (95% CI) were calculated. In the presence of heterogeneity (I2 > 50%), a random‐effect model was used. Otherwise, a fixed‐effect model was used. Statistical significance was defined by *p value of* <0.05. Meta‐analysis was performed using Cochrane RevMan (v.5.4, 2020; Cochrane Initiative). The quality of our effect estimates was assessed using the Grading of Recommendations, Assessment, Development and Evaluations (GRADE) rating system.^23^

## 3 RESULTS

### 3.1 Study characteristics

Our search identified 13 894 articles of which 1214 had their abstracts screened. A total of 1173 abstracts were excluded leaving 41 full text articles. Based upon our eligibility criteria, a further 22 articles were excluded, leaving a total of 19 studies included from the database searches. A further two articles were included from searching references of included articles, leaving 21 studies included in the present review^9^, ^24^, ^25^, ^26^, ^27^, ^28^, ^29^, ^30^, ^31^, ^32^, ^33^, ^34^, ^35^, ^36^, ^37^, ^38^, ^39^, ^40^, ^41^, ^42^, ^43^ (Figure 3).

Table 1 shows the study characteristics. Eighteen studies were observational. Eight of the studies were prospective, of which three were RCTs,^24^, ^40^, ^41^ three were cohort studies^27^, ^28^, ^36^ and two were case series.^35^, ^42^ Thirteen studies were retrospective, comprising 10 cohort studies^25^, ^26^, ^29^, ^32^, ^33^, ^34^, ^37^, ^38^, ^39^, ^43^ and 3 case series.^9^, ^30^, ^31^ The mean or median follow‐up durations were reported in all but one study^41^ (Table 1).

**TABLE 1:** Baseline characteristics of all included studies

Study ID (author and country)	Type of study		Recruitment period	Type of surgery	Number of patients			Follow up duration *(median in months unless stated)*	
					entire study	OSC	SRC	OSC	SRC
Jacobs et al., USA^21^	RCT	Prospective	2007–2011	Capsule sparing vs. nerve sparing	40	CS: 20 NS: 20	0	CS = 41 NS = 37	N/A
Basiri et al., Iran^22^	Cohort	Retrospective	2003–2008	Prostate sparing vs. SRC	50	23	27	Mean = 39	Mean = 35
Vilaseca et al., Spain^23^	Cohort	Retrospective	2006–2009	Nerve sparing vs. SRC	44	11	33	21 (NR if mean or median)	‐
El‐Bahnasawy et al., Egypt^24^	Cohort	Prospective	2003–2005	Nerve sparing vs. SRC	60	30	30	38.8 ± 19.2 (NR if mean or median)	42.9 ± 26.9 (NR if mean or median)
Kessler et al., Switzerland^25^	Cohort	Prospective	1985–2003	Nerve sparing vs. SRC	331	256	75	Overall Median = 31.2	
Colombo et al., Italy^26^	Cohort	Retrospective	1997–2012	Capsule sparing vs. seminal sparing vs. nerve sparing	90	CS: 36 SS: 19NS: 35	0	Mean NS = 111.71 Mean CS = 133.69 Mean SS = 43.15	N/A
*Rozet et al., France^27^	Case series	Retrospective	1992–2004	Capsule sparing	108	108	0	Mean = 55 ± 3.6	N/A
*Muto et al., Italy^28^	Case series	Retrospective	1990–2009	Seminal sparing	91	91	0	Mean = 102	N/A
Chen and Chiang, Taiwan^29^	Cohort	Retrospective	2007–2015	Prostate sparing vs. SRC	25	14	11	Mean = 51.14	Mean = 73.82
**Bai et al., China** ^30^	Cohort	Retrospective	2007–2017	Uterus and ovary sparing vs. SRC	90	45	45	34	38
Furrer et al., Switzerland^31^	Cohort	Retrospective	1985–2007	Nerve sparing vs. SRC	180	156	24	Unilat. NS = 174 Bilat. NS = 163	177
*Gross et al., Switzerland ^32^	Cohort	Prospective	‐	Nerve sparing	73	73	0	Mean = 64	N/A
*Vallancien et al. France^7^	Case series	Retrospective	1992–2002	Prostate sparing	100	100	0	Mean = 38	N/A
*Voskuilen et al., Netherlands and France^33^	Case series	Prospective	1995–2014	Prostate sparing	185	185	0	90	N/A
**Bhatt et al., USA** ^34^	Cohort	Retrospective	2002–2004	Nerve sparing vs. SRC	13	6	7	Mean = 13.2	Mean = 27.3
Saad et al., France^35^	Cohort	Retrospective	2001–2012	Prostate‐sparing vs. Nerve‐sparing	107	PS: 60 NS: 47	0	PS = 73 NS = 62	N/A
**Huang et al., China** ^36^	Cohort	Retrospective	2006–2017	Uterus‐sparing vs. SRC	112	63	49	36	36
Moussa et al., Lebanon^37^	RCT	Prospective	2014–2019	Nerve Sparing vs. SRC	160	80	80	12	12
Abdelaziz et al., Egypt^38^	RCT	Prospective	2014–2016	Capsule Sparing vs. SRC	96	45	51	‐	N/A
Mertens et al., Netherlands^39^	Case Series	Prospective	1994–2013	Prostate sparing	110	110	0	77	N/A
De Vries et al,. Netherlands^40^	Cohort	Retrospective	1994–2006	Prostate sparing vs SRC	126	63	63	56	76

OSC = Organ‐Sparing Cystectomy; SRC = Standard Radical Cystectomy; IQR = interquartile range; CS = capsule sparing; SS = seminal sparing; NS = nerve sparing; UOS = Uterus and Ovary Sparing; RCT = Randomised Controlled Trial; **BOLD = Female‐Only Studies**; * = Single arm studies; − = not reported; N/A = Not Applicable.

The patient selection and inclusion criteria varied between the identified papers, and this information is summarised in Table 2. Normal preoperative sexual function was recorded in 11 studies^26^, ^27^, ^29^, ^31^, ^34^, ^36^, ^37^, ^38^, ^39^, ^40^, ^42^, ^43^ (Table 2).

**TABLE 2:** Patient selection in all included studies

Study ID	Inclusion criteria							
	Tumour‐free (bladder neck/prostatic urethra)	Absence of prostate cancer				Organ‐confined disease	Normal preoperative sexual function	Age
		DRE	PSA	Biopsy (TRUS)	Biopsy (TURP)			
***Nerve sparing***								
**Vilaseca et al.** ^23^	**‐**	**‐**	**‐**	**‐**	**‐**	**‐**	**✓**	**✓**
**El‐Bahnasawy et al.** ^24^	**✓**	**‐**	**‐**	**‐**	**‐**	**✓**	**✓**	**✓**
**Kessler et al.** ^25^	**‐**	**‐**	**‐**	**‐**	**‐**	**✓**	**‐**	**‐**
**Furrer et al.** ^31^	**‐**	**‐**	**‐**	**‐**	**‐**	**✓**	**✓**	
*Gross et al. ^32^	**‐**	**‐**	**‐**	**‐**	**‐**	**‐**	**‐**	
**Bhatt et al.** ^34^	**✓**	**‐**	**‐**	**‐**	**‐**	**✓**	**✓**	**✓**
**Moussa et al.** ^37^	**‐**	**‐**	**‐**	**‐**	**‐**	**✓**	**✓**	**‐**
***Prostate sparing***								
*Vallancien et al. ^7^	**✓**	**✓**	**✓**	**✓**	**✓**	**‐**	**‐**	**‐**
**Basiri et al.** ^22^	**✓**	**✓**	**✓**	**‐**	**✓**	**‐**	**‐**	**‐**
**Chen and Chiang** ^29^	**✓**	**✓**	**✓**	**‐**	**‐**	**‐**	**‐**	**‐**
*Voskuilen et al. ^33^	**✓**	**✓**	**✓**	**✓**	**✓**	**‐**	**✓**	**‐**
**Mertens et al.** ^39^	**✓**	**✓**	**✓**	**✓**	**‐**	**✓**	**✓**	**‐**
**De Vries et al.** ^40^	**✓**	**✓**	**✓**	**✓**	**✓**	**✓**	**✓**	**‐**
***Seminal‐vesicle sparing***								
*Muto et al. ^28^	**✓**	**✓**		**✓**	**✓**	**✓**	**✓**	**✓**
***Uterus and ovary sparing***								
**Bai et al.** ^30^	**‐**	**N/A**	**N/A**	**N/A**	**N/A**	**✓**	**‐**	**✓**
**Huang et al.** ^36^	**✓**	**N/A**	**N/A**	**N/A**	**N/A**	**‐**	**‐**	**✓**
***Capsule sparing***								
*Rozet et al. ^27^	**✓**	**✓**	**✓**	**✓**	**✓**	**‐**	**‐**	**‐**
**Abdelaziz et al.** ^38^	**✓**	**✓**	**✓**	**✓**	**✓**	**✓**	**‐**	**✓**
***Sparing* vs. *sparing***								
**Jacobs et al.** ^21^	**✓**	**‐**	**✓**	**✓**	**✓**	**✓**	**‐**	**‐**
**Colombo et al.** ^26^	**✓**	**✓**	**✓**	**✓**	**✓**	**✓**	**✓**	**✓**
**Saad et al.** ^35^	**✓**	**‐**	**✓**	**✓**	**✓**	**✓**	**✓**	**✓**

SRC = Standard Radical Cystectomy; DRE = Digital Rectal Examination, PSA = Prostate‐Specific Antigen; TRUS = Transrectal Ultrasound Guided; TURP = Transurethral Resection of Prostate; **BOLD = Female‐Only Studies;** * = Single Arm Studies; − = Not Recorded; N/A = Not Applicable.

In total, 1886 male patients were included across 17 studies. Eight studies reported on nerve‐sparing cystectomy,^24^, ^26^, ^27^, ^28^, ^29^, ^34^, ^38^, ^40^ seven on prostate‐sparing cystectomy,^9^, ^25^, ^32^, ^36^, ^38^, ^42^, ^43^ four on capsule‐sparing cystectomy^24^, ^29^, ^30^, ^41^ and two on seminal vesicle‐sparing cystectomy.^29^, ^31^

There were a total of 305 female patients included across five studies^33^, ^34^, ^35^, ^37^, ^39^; four of which were female‐only studies.^33^, ^35^, ^37^, ^39^ Three of these studies reported on nerve‐sparing cystectomy,^33^, ^34^, ^35^, ^37^ whereas two studies also documented uterus sparing and uterus, vagina, ovary and fallopian tube‐sparing techniques, respectively.^33^, ^39^

### 3.2 Oncological outcomes

Table 3 summarises the clinical and pathological characteristics of participants in 19 studies, with two studies not reporting any data pertaining to this.^33^, ^40^

**TABLE 3:** Oncological outcomes: clinical and pathological characteristics of participants in all included studies

Study ID	Clinical bladder stage (>T2/N+) (*%*)			Pathological bladder stage (>T2/N1) (*%*)			Incidental prostate cancer (%)			
	OSC	SRC	*p*	OSC	SRC	*p*	OSC	SRC	*p*	GS ≥ 8
***Nerve sparing***										
Vilaseca et al.^23^	>T2: 0	>T2: 0	N/S	N+: 15.9	‐	‐	‐	‐	‐	‐
El‐Bahnasawy et al.^24^	>T2: 0	>T2: 0	N/S	>T2: 52.3 N+: 9.5	>T2: 54.1 N1: 16.7	0.42 0.42	‐	‐	‐	‐
Kessler et al.^25^	N+: 0	N+: 0	‐	>T2: 51.7 N+: 23.9	‐	‐	‐	‐	‐	‐
Furrer et al.^31^	‐	‐	‐	>T2: 27 N1: 26	>T2: 29 N1:8	‐	‐	‐	‐	‐
**Bhatt et al.** ^34^	>T2: 0	>T2: 0	‐	>T2: 17	>T2: 0	‐	‐	‐	‐	‐
***Prostate sparing***										
*Vallancien et al.^7^	>T2: 36	‐	‐	>T2: 23 N+: 13	‐	‐	22	‐	‐	‐
Basiri et al.^22^	>T2: 17.4 N+: 0	>T2: 3.7 N+:0	0.31	>T2: 26.0	>T2: 33.3	0.51	4.3	7.4	0.86	0
Chen and Chiang^29^	‐	‐	‐	>T2/N+: 36.0	>T2/N+: 20.0	0.351	‐	‐	‐	‐
*Voskuilen et al.^33^	>T2: 10.3 N+: 13	‐	‐	>T2: 22.7 N+: 35	‐	‐	3.2	‐	‐	‐
Mertens et al.^39^	>T2: 17 (15.3%) cT1‐4 N0–3	‐	‐	‐	‐	‐	3 (2.7%)	‐	‐	‐
De Vries et al.^40^	>T2: 15.9 N+: 11.1	>T2: 23.8 N+: 11.1	N/S	>T2: 25.4 N+: 28.6	>T2:34.9 N+: 19	N/S	3.2	14.3	‐	‐
***Seminal‐vesicle sparing***										
*Muto et al.^28^	>T2: 0 N+: 0	‐	‐	>T2: 1.1 N1: 0	‐	‐	2.2	‐	‐	‐
***Uterus and ovary sparing***										
**Bai et al.** ^30^	‐	‐	‐	>T2: 18 N+: 68	>T2: 11 N1: 6	0.951	N/A	N/A	N/A	N/A
**Huang et al.** ^36^	>T2: 30.2	>T2: 42.9	‐	>T2: 36.5	>T2: 40.8	‐	N/A	N/A	N/A	N/A
***Capsule sparing***										
*Rozet et al.^27^	‐	‐	‐	>T2: 40.7 N1: 13.9	‐	‐	0.08	‐	‐	‐
Abdelaziz et al.^38^	>T2: 0	>T2: 0	‐	‐	‐	N/S	0	0	‐	‐
***Sparing* vs. *sparing***										
Jacobs et al.^21^ *‐Capsule sparing (CS)* *‐Nerve sparing (NS)*	CS: T2 + T3: 50.0 N+: 0 NS: T2 + T3: 70.0 N+: 0	N/A	0.23	CS: >T2: 10.0 N+: 10.0 NS: >T2: 20.0 N1: 25.0	N/A	0.45 0.19	CS: 15 NS: 40	N/A	0.15	0
Colombo et al.^26^ *‐Capsule sparing (CS)* *‐Nerve sparing (NS)* *‐Seminal vesicle sparing (SS)*	>T2: 0	N/A	N/S	CS: >T2: 8.3 N+: 2.8 NS: >T2: 8.6, N+: 2.9 SS: >T2: 5.3, N+: 0	N/A	>T2: 0.5 N+: 0.4	NS: 17.1 SS: 15.8	N/A	‐	‐
Saad et al.^35^ *‐Prostate sparing (PS)* *‐ Nerve sparing (NS)*	PS: >T2: 5 NS: >T2: 17	N/A	0.5	PS: >T2: 22 N+: 8 NS: >T2: 23 N+: 19	N/A	0.08	PS: 3 NS: 45	N/A	<0.001	‐

OSC = Organ‐Sparing Cystectomy; SRC = Standard Radical Cystectomy; GS = Gleason Score; CS: capsule sparing; NS = nerve sparing; SS = seminal sparing; BOLD = Female‐Only Studies; * = Single‐Arm Studies; − = Not Recorded; N/A = Not Applicable; N/S = Not Significant.

Table 4 highlights the recurrence‐free survival, progression‐free survival, disease‐specific survival and overall survival rates reported in 13 studies. One of the comparative prostate‐sparing studies reported a 5‐year overall survival rate of 47% compared to 30% in the radical technique (*p* > 0.05).^25^ Another study found overall survival rates of 67% and 83%, when comparing uterus and ovary‐sparing technique to standard RC, respectively (*p* = 0.074); although, the time‐point was not reported.^33^ There was no data available pertaining to the site or timing of recurrence (Table 4).

**TABLE 4:** Oncological outcomes: recurrence‐free survival, progression‐free survival, disease‐specific survival and overall survival

Study ID	Recurrence‐free survival (%)			Progression‐free survival (%)			Disease‐specific survival (%)			Overall survival (%)		
	OSC	SRC	*p* value	OSC	SRC	*P* value	OSC	SRC	*p* value	OSC	SRC	*p* value
***Prostate sparing***												
*Vallancien et al.^7^	63 (pT3) 8 (N‐)	‐	‐	47 (grade III tumours)	‐	‐	‐	‐	‐	pT3: 71 N‐: 54	‐	‐
Basiri et al.^22^	‐	‐	‐	‐	‐	‐	5 Yr: 35	5 Yr: 13	‐	5 Yr: 47	5 Yr: 30	0.05
Chen and Chiang^29^	0.7 (Kaplan–Meier survival curve)	0.8 (Kaplan–Meier survival curve)	0.738	‐	‐	‐	‐	‐	‐	‐	‐	‐
*Voskuilen et al.^33^	‐	‐	‐	‐	‐	‐	‐	‐	‐	5 Yr: 70.6	‐	‐
Mertens et al.^39^	2 Yr: 71.2 5 Yr: 66.6	‐	‐	‐	‐	‐	2 Yr: 76.2 5 Yr: 66.5	‐	‐	2 Yr: 74.4 5 Yr: 64.2	‐	‐
De Vries et al.^40^	‐	‐	‐	‐	‐	‐	5 Yr: 66%	5 Yr: 64%	0.6	‐	‐	‐
***Seminal‐vesicle sparing***												
*Muto et al.^28^	‐	‐	‐	6 Yr CSS: 90.2–93.3	‐	‐	6 Yr CSS: 88.5–93.3	‐	‐	6 Yr CSS: 89.6–93.3	‐	‐
***Uterus and ovary sparing (UOS)***												
**Bai et al.** ^30^	70	76	0.11	‐	‐	‐	75	86	0.071	67	83	0.074
**Huang et al.** ^36^	‐	‐	‐	5Yr: 69.4	5 Y: 77.7	0.565	‐	‐	‐	5Yr: 59.6	5Yr: 62.2	0.939
***Capsule sparing***												
*Rozet et al.^27^	‐	‐	‐	‐	‐	‐	5 Yr: 71	‐	‐	5 Yr: 67	‐	‐
Abdelaziz et al.^38^	2 Yr: 97.9	2 Yr: 91.1	0.15	‐	‐	‐	2 Yr: 97.9	2Yr: 96.4	0.65	2 Yr: 93.7	2 Yr: 91.1	0.62
***Sparing* vs. *sparing***												
Jacobs et al.^21^ *‐Capsule sparing (CS)* *‐Nerve sparing (NS)*	‐	‐	>0.05	‐	‐	>0.05	‐	‐	‐	‐	‐	>0.05
Saad et al.^35^ *‐Prostate sparing (PS)* *‐Nerve sparing (NS)*	‐	‐	‐	‐	‐	‐	5 Yr PS: 90 5 Yr NS: 78	‐	0.055	‐	‐	‐

OSC = Organ‐Sparing Cystectomy; SRC = Standard Radical Cystectomy; CS: capsule sparing; NS = nerve sparing; SS = seminal sparing; UOS = Uterus and Ovary Sparing; CSS = Cancer Specific Survival; mo = months BOLD = Female‐Only Studies; * = Single‐Arm Studies; IQR = Interquartile Range; R = Range; − = Not Reported; N/A = Not Applicable.

Table 5 presents the 10 studies that reported local and metastatic recurrence rates. One study that compared three techniques, capsule‐sparing, nerve‐sparing and seminal vesicle‐sparing techniques, reported non‐significantly different local recurrence rates of 5.5%, 5.7% and 0%, respectively (*p* = 0.5)^26^ (Table 5).

**TABLE 5:** Oncological outcomes: local recurrence and metastatic disease

Study ID	Local recurrence *(%)*			Metastatic disease *(%)*		
	OSC	SRC	*p* value	OSC	SRC	*p* value
***Nerve sparing***						
Vilaseca et al.^23^	18.1	21.2	0.14	‐	‐	‐
***Prostate sparing***						
*Vallancien et al.^7^	5.1	‐	‐	31.6	‐	‐
Basiri et al.^22^	61.1	55.0	> 0.05	‐	‐	‐
*Voskuilen et al.^33^	6.5	‐	‐	23.8	‐	‐
Mertens et al.^39^	11	‐	‐	38	‐	‐
De Vries et al.^40^	7.9	15.9	0.1	28.6	33.3	0.4
***Seminal‐vesicle sparing***						
*Muto et al.^28^	2.2	‐	‐	6.6	‐	‐
***Uterus and ovary sparing***						
**Bai et al.** ^30^	‐	‐	‐	17.3	27.5	0.17
***Capsule sparing***						
*Rozet et al.^27^	4.6	‐	‐	33.3	‐	‐
***Sparing* vs. *sparing***						
Colombo et al.^26^ *‐Capsule sparing (CS)* *‐Nerve sparing (NS)* *‐Seminal vesicle sparing (SS)*	CS: 5.5 NS: 5.7 SS: 0	‐	0.5	‐	‐	‐

OSC = Organ‐Sparing Cystectomy; SRC = Standard Radical Cystectomy; GS = Gleason Score; CS = capsule sparing; NS = nerve sparing; SS = seminal sparing; **BOLD = Female‐Only Studies;** * = Single‐Arm Studies; − = Not Recorded.

In total, only four studies included comparative data suitable for meta‐analysis of oncological outcomes, which were divided into subgroups: local recurrence and metastatic disease. All three of the studies used in the meta‐analysis of local recurrence were male only.^25^, ^26^, ^43^ In contrast, one of the two studies used in the meta‐analysis of metastatic disease included exclusively females.^33^

**FIGURE 4:**
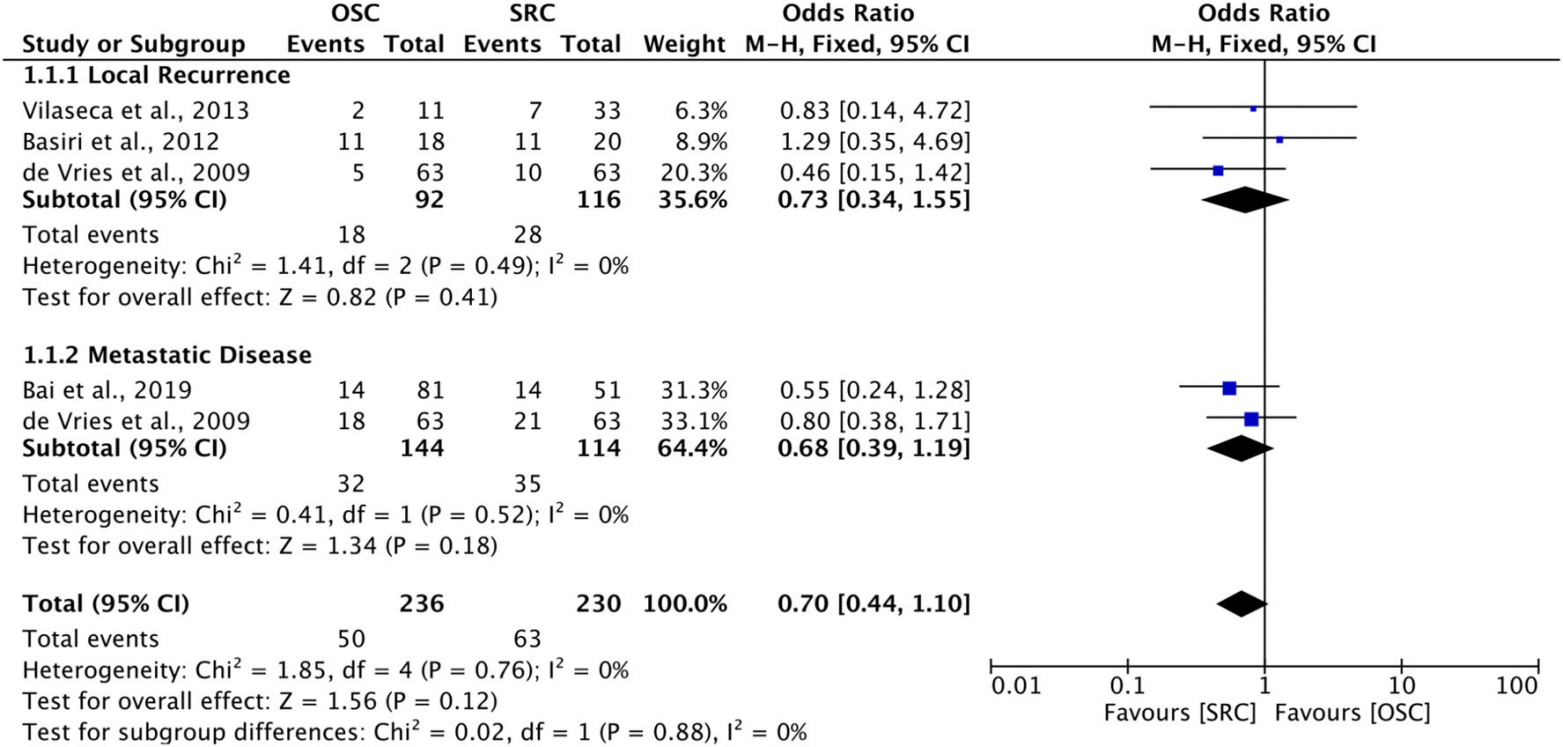
Meta‐analysis of local and metastatic recurrence rates

The meta‐analysis of local recurrence rates featured three studies, two analysing prostate‐sparing techniques and the other nerve‐sparing technique.^25^, ^26^, ^43^ There were similar local recurrence rates between organ‐sparing and RC papers (OR 0.73; 95% CI 0.34–1.55, *p* = 0.41). The meta‐analysis of metastatic disease featured two studies and showed similar rates of metastatic recurrence between OSC and RC (OR 0.68; 95% CI 0.30–1.19, *p* = 0.18).^33^, ^43^ In terms of meta‐analysis of overall recurrence, when both local and metastatic data from these four studies were pooled, the overall recurrent rate was similar between organ‐sparing and RC patients (OR 0.70; 95% CI 0.44–1.10, *p* = 0.12)^25^, ^26^, ^33^, ^43^ (Figure 4).

### 3.3 Sexual outcomes

Male sexual function was evaluated in 13 studies, as highlighted in Table 6.^9^, ^24^, ^25^, ^26^, ^27^, ^29^, ^31^, ^32^, ^36^, ^38^, ^40^, ^41^, ^42^ To assess potency, the primary functional outcome, seven studies used the IIEF‐5 questionnaire. However, other means of assessing potency, such as the Erectile Hardness Score (EHS), Bladder Cancer Index (BCI) and a self‐designed questionnaire were also used in one study each. Three studies did not explicitly state how patients' potency was assessed.^9^, ^36^, ^42^

**TABLE 6:** Male sexual function outcomes

Study ID	Postoperative sexual function assessment				Sexual outcomes ‐ potency (%)			
	Timeframe (mo)	Questionnaire	Self‐impression	Number of patients **evaluated (*n*)**	OSC	SRC	*p* value	Treatment of ED (%)
***Nerve sparing***								
Vilaseca et al.^23^	‐	EHS	No	SRC: 33 NS: 11	77.7	4.5	<0.001	Intervention: 100, control (PDE‐5): 23
El‐Bahnasawy et al.^24^	12	IIEF‐5	No	SRC: 24 NS: 21	80.95	0	<0.05	intervention: 23 (PDE‐5)
Chen and Chiang^29^	12	IIEF‐5	Yes	SRC: 7 NS: 10	100	0	0.014	‐
Moussa et al.^37^	12	IIEF‐5	No	SRC:80 NS:80	Preoperative ‐ 21.4 Postoperative ‐ 20.9	Preoperative ‐ 20.7 Postoperative ‐ 10.8	‐	**4 predesigned subgroups: 1‐ None 2 ‐ PGE‐1 3 ‐ PGE − 1 + PDE‐5 4 ‐ PDE‐5
***Prostate sparing***								
*Voskuilen et al.^33^	‐	‐	Yes	PS: 185	88.90	‐	0.483	24.3 (19.9 sildenafil and 4.4 intracavernous injections)
Basiri et al.^22^	6	IIEF‐5	Yes	SRC: 12 PS: 12	83	17	0.002	‐
*Vallancien et al.^7^	12	‐	Yes	PS: 61	82	‐	‐	‐
Mertens et al.^39^	‐	‐	Yes	87 (79.1%)	89.7 (of those evaluated)	‐	‐	‐
***Capsule sparing***								
Abdelaziz et al.^38^	12	IIEF‐5	No	SRC: 40 CS: 41	97.6	0	<0.001	‐
***Seminal‐vesicle sparing***								
*Muto et al.^28^	6	IIEF‐5	No	SS: 91	100	‐	‐	4.4 (1 Oral therapy and 3.3 Alprostadil)
***Sparing* vs. *sparing***								
Colombo et al.^26^ *‐Capsule sparing (CS)* *‐Nerve sparing (NS)* *‐Seminal vesicle sparing (SS)*	24	IIEF‐5	Yes	CS: 36 NS: 35 SS: 19	CS: 91.6 NS: 28.6 SS: 84.2	‐	<0.01	PDE‐5 CS: 19.4 NS: 54.2 SS: 31
Jacobs et al.^21^ *‐Capsule sparing (CS)* *‐Nerve sparing (NS)*	12	Yes, BCI	Yes	CS: 20 NS: 20	CS: 50 NS: 45	‐	0.06	PDE‐5 CS: 55 NS: 65
Saad et al.^35^ *‐Prostate sparing (PS)* *‐ Nerve sparing (NS)*	NR (best sexual function at any time‐point used)	Self‐designed	Yes	PS: 48 NS: 29	PS: 66.7 NS: 13.8	‐	<0.001	PS PDE‐5: 16.7 Intracavernosal injection: 16.7 Prosthesis: 0 NS PDE‐5: 0 Intracavernosal injection: 72.4 Prosthesis: 13.8

OSC = Organ‐Sparing Cystectomy; SRC = Standard Radical Cystectomy; CS: Capsule Sparing; NS = Nerve Sparing; SS = Seminal Sparing; CSS = Cancer Specific Survival; BCI = Bladder Cancer Index, IIEF‐5 = International Index Erectile Function 5; mo. = months; PDE‐5 = Phosphodiesterase‐5; EHS = Erectile Hardness Score; ED = Erectile Dysfunction; * = Single‐Arm Studies; ** = Extra Information ‐ = Not Recorded; N/A = Not Applicable.

**FIGURE 5:**
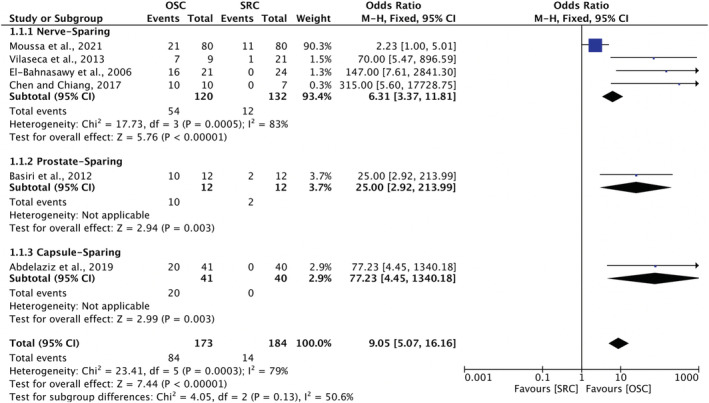
Meta‐analysis of male potency rates

Of the aforementioned studies, only five had data suitable for comparison in a meta‐analysis. All five studies analysed male patients only. Four of the studies compared nerve sparing^26^, ^27^, ^32^, ^40^, whereas the fifth compared prostate sparing.^25^ The patients undergoing an OSC had significantly better postoperative potency outcomes of 80.95–100% compared to 0–17% in the RC group. One study reported a reduction from preoperative potency to postoperative potency of 0.5% in nerve‐sparing cystectomy compared to a greater reduction of 9.9% in standard RC.^40^ The meta‐analysis suggests organ‐sparing techniques are associated with superior outcomes in terms of retaining potency (OR 9.05; 95% CI 5.07–16.16, *p* < 0.00001) (Figure 5).

Three studies compared different organ‐sparing techniques with regards to potency rates. The first two studies analysed capsule‐sparing and nerve‐sparing cystectomies.^24^, ^29^ The second of these papers also compared these potency outcomes to a third cohort of patients undergoing seminal vesicle sparing.^29^ The patients who underwent capsule‐sparing cystectomies had a 50–91.6% potency rate, in comparison to the nerve‐sparing patients, who had a 28.6–45% potency rate (*p* < 0.01). The patients undergoing a seminal vesicle‐sparing cystectomy had an 84.2% potency rate (*p* = 0.06). The third study reported a significantly greater potency rate of 66.7% in prostate‐sparing cystectomy compared to 13.8% in nerve‐sparing cystectomy (*p* < 0.001)^38^ (Table 6).

In total, seven studies reported on the use of adjuvant treatments to aid sexual rehabilitation. The percentage of patients who required treatment to restore sexual function ranged from 1 to 100%, as defined by the trialists.^24^, ^26^, ^29^, ^31^, ^36^, ^38^, ^40^ All these patients were considered to have maintained potency status. There was no relevant data regarding patients' fertility or capability to orgasm (Table 6).

As there was only one paper studying sexual outcomes in female patients, no meta‐analysis could be performed. This study compared the postoperative sexual function in 13 females undergoing either a nerve‐sparing or RC using the Female Sexual Function Index questionnaire.^37^ Those who underwent a nerve‐sparing cystectomy averaged a score of 22.3 compared to 11 for RC. There was no comment made towards patients' vaginal length, ability to have penetrative sex or use of hormone‐replacement therapy (Table 7).

**TABLE 7:** Female sexual function outcomes

Study ID	Postoperative sexual function assessment				Sexual outcomes ‐ FSFI mean composite score		
	Timeframe (mo)	Questionnaire	Self‐impression	Number of patients evaluated (*n*)	OSC	SRC	*p* value
***Nerve sparing***							
**Bhatt et al.** ^34^	12	FSFI	No	SRC: 7 NS: 6	22.3	11	‐

OSC = Organ‐Sparing Cystectomy; SRC = Standard Radical Cystectomy; NS = nerve‐sparing; FSFI = Female Sexual Function Index; mo = months; **BOLD = Female‐Only Studies;** − = Not Recorded; N/A = Not Applicable.

### 3.4 Urinary outcomes

Fourteen studies analysed continence rates using several methods, including questionnaires such as the BCI, and number of pads. Twelve studies reported 100% of patients having orthotopic neobladder formation.^9^, ^24^, ^25^, ^27^, ^28^, ^31^, ^32^, ^34^, ^35^, ^38^, ^41^, ^42^ One study reported 86.4% of patients having an orthotopic neobladder and the remainder 13.6% of patients having an ileal conduit, with continence outcomes only reported in the patients with orthotopic neobladders.^26^ One study did not specify the type of urinary diversion.^29^

**FIGURE 6:**
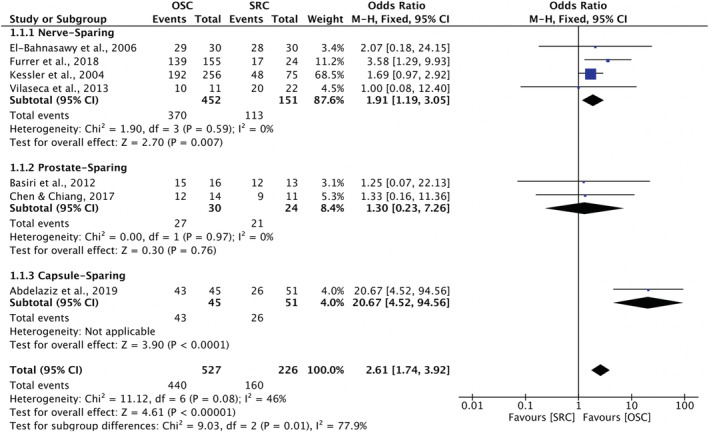
Meta‐analysis of daytime continence rates

**FIGURE 7:**
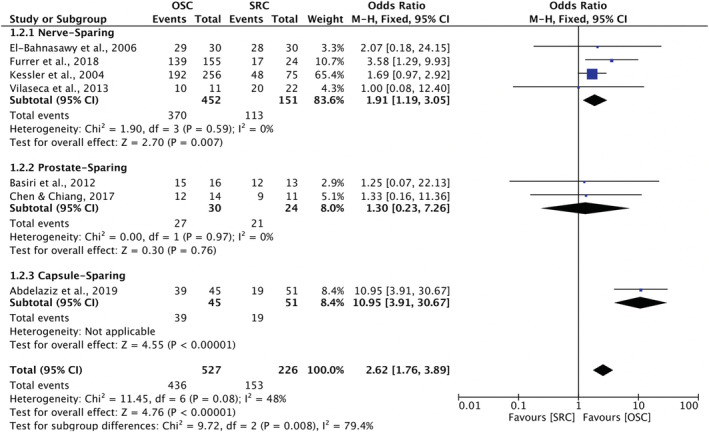
Meta‐analysis of nighttime continence rates

Table 6 presents the reported daytime and nighttime continence rates in the 14 studies. Seven of these studies had comparative male data suitable for meta‐analysis, both for daytime continence and nighttime continence. Four reported on nerve‐sparing cystectomy,^26^, ^27^, ^28^, ^34^ two on prostate‐sparing cystectomy^25^, ^32^ and one study compared capsule‐sparing cystectomy to standard RC.^41^ Regarding daytime continence, meta‐analysis showed that OSC was associated with significantly better daytime continence compared with RC (OR 2.61; 95% CI 1.74 to 3.92, *p* < 0.00001) (Figure 6). Similarly, regarding nighttime continence, meta‐analysis also showed OSC was associated with better nighttime continence compared to RC (OR 2.62; 95% CI 1.76 to 3.89, *p* < 0.00001) (Figure 7).

Only one study analysed continence in female patients, observing continence rates of 60.9% in nerve‐sparing cystectomy patients compared to 25% in standard RC patients. The study used self‐impression to measure continence and did not indicate whether this was daytime or nighttime continence^35^ (Table 8).

**TABLE 8:** Urinary function outcomes

Study ID	Timeframe (*months*)	Measurement of continence	Number of patients evaluated (*n*)	Daytime continence (%)			Nighttime continence (%)		
				OSC	SRC	*p* value	OSC	SRC	*p* value
***Nerve sparing***									
Kessler et al.^25^	12	Standardised questionnaire	SRC: 75 NS: 256	75.0	64.0	0.002	70.0	56.0	0.036
El‐Bahnasawy et al.^24^	12	Standardised questionnaire	SRC: 30 NS: 30	96.7	93.3	‐	70.0	63.3	>0.05
Vilaseca et al.^23^	NR	Number of Pads	SRC: 22 NS: 11	90.9	90.9	0.999	54.5	50.0	0.805
Furrer et al.^31^	12	ICIQ‐UI SF and Number of Pads	SRC: 24 NS: 155	89.7	70.8	‐	67.7	54.2	‐
^*Gross et al. ^32^	NR	Self‐impression	SRC: 4 NS: 69	60.9	25	‐	‐	‐	‐
***Prostate sparing***									
Chen and Chiang^29^	12	Number of Pads	SRC: 11 PS: 14	85.7	81.8	0.754	64.2	54.5	0.746
Basiri et al.^22^	> 6	Number of Pads, reported to physician	SRC: 13 PS: 16	93.8	92.3	N/S	81.3	61.5	N/S
*Vallencien et al.^7^	12	Mailed questionnaires	PS: 88	96.6	‐	‐	95	‐	‐
Mertens et al.^39^	‐	interview on voiding, continence and pad use	105 (95.5%)	complete: 96.2 satisfactory: 3.8	‐	‐	complete: 81.9 satisfactory: 13.3 poor: 4.8	‐	‐
***Capsule sparing***									
Abdelaziz et al.^38^	12	Number of pads	SRC: 51 CS: 45	95.6	51	<0.001	86.7	37.3	<0.001
***Seminal vesicle sparing***									
*Muto et al.^28^	NR	Voiding Diary	SS: 91	96	‐	‐	37	‐	‐
***Sparing* vs. *sparing***									
Colombo et al.^26^ *‐Capsule sparing (CS)* *‐Nerve sparing (NS)* *‐Seminal vesicle sparing (SS)*	24	Number of Pads	CS: 36 NS: 35 SS: 19	CS: 97.2 NS: 88.6 SS: 94.7	N/A	0.3	CS: 83 NS: 57 SS: 63	N/A	0.05
**^**Jacobs et al.^21^ *‐Capsule sparing (CS)* *‐Nerve sparing (NS)*	12	BCI Questionnaire (pts reduction from baseline)	CS: 20 NS: 20	CS: 13 ± 30 points NS: 28 ± 33 points	N/A	‐	‐	N/A	‐
Saad et al.^35^ *‐Prostate sparing (PS)* *‐ Nerve sparing (NS)*	12	Questionnaire inc number of pads	PS: 59 DC 54 NC NS: 46 DC 38 NC	PS: 91.5 NS: 52.2	N/A	<0.001	PS: 77.8% NS: 21.1%	N/A	<0.001

OSC = Organ‐Sparing Cystectomy; SRC = Standard Radical Cystectomy; CS: capsule sparing; NS = nerve sparing; SS = seminal sparing; BCI = Bladder Cancer Index; ICIQ‐UI SF = International Consultation on Incontinence Questionnaire‐Urinary Incontinence Short Form; **BOLD** = Female‐Only Studies; * = Single‐Arm Studies; ^ = Study does not specify nighttime or daytime continence; − = Not Recorded; N/A = Not Applicable; N/S = Not Significant.

Three studies examined the difference in continence rates between various types of OSC.^24^, ^29^, ^38^ One of the studies reported a greater daytime continence rate of 97.2% in capsule‐sparing cystectomy, compared to 94.7% in seminal vesicle‐sparing cystectomy and 88.6% in nerve‐sparing cystectomy. Similarly, a greater nighttime continence of 83% was reported in capsule‐sparing cystectomy patients, compared to 63% of seminal vesicle‐sparing cystectomy patients and 57% of nerve‐sparing cystectomy patients.^29^ In the other study, the authors observed a reduction in average urinary function of 13 ± 30 points from BCI questionnaire baseline in capsule sparing cystectomy, compared to a reduction of 28 ± 33 points in nerve‐sparing cystectomy (*p* = 0.1).^24^ The third study reported greater daytime and nighttime continence rates of 91.5% and 77.8%, respectively, in prostate‐sparing cystectomy compared to 52.2% and 21.1% in nerve‐sparing cystectomy, respectively (*p* < 0.001)^38^ (Table 8).

Only one study performed urodynamic assessment, showing better urodynamic outcomes in male patients who had nerve‐sparing cystectomy compared to RC. The study observed a significantly longer functional urethral length of 34.8 mm in the nerve‐sparing cystectomy group, compared to a length of 30.1 mm in the RC group. Furthermore, the study reported a greater maximum urethral pressure of 82.8 cm H20 in the nerve‐sparing cystectomy group, compared to 77.9 cm H20 in the RC group.^27^

### 3.5 Risk of bias assessment

The data regarding the risk of bias assessment is detailed in Tables 9, 10, 11. Regarding the three RCTs included, risk of bias was rated as low risk in one study,^41^ and there were some concerns with the other two studies^24^, ^40^ (Table 9). Moderate risk of bias was found for 12 of the non‐randomised comparative studies,^25^, ^26^, ^27^, ^28^, ^29^, ^32^, ^33^, ^34^, ^35^, ^38^, ^39^, ^43^ with serious risk of bias found for one^37^ (Table 10). Of the five case series included, there were some concerns with three,^9^, ^31^, ^37^ whereas two were deemed low risk^30^, ^42^ (Table 11).

**TABLE 9:** Risk of bias for RCTs as measured by the Cochrane Risk of Bias 2.0 tool^17^

Study ID	Randomization process	Deviations from intended interventions	Missing outcome data	Measurement of the outcome	Selection of the reported result	Overall
Jacobs et al.^21^	Low risk	Low risk	Unclear	Unclear	Low risk	Some concerns
Moussa et al.^37^	Low risk	Low risk	Low risk	Low risk	Some concerns	Some concerns
Abdelaziz et al.^38^	Low risk	Low risk	Low risk	Low risk	Low risk	Low risk

**TABLE 10:** Risk of bias for non‐randomised comparative studies using the ROBINS‐I tool^18^

Study ID	Preintervention		At intervention	Postintervention				Overall risk of bias
	Bias because of confounding	Bias in selection of participants into the study	Bias in classification of interventions	bias because of deviations from intended interventions	Bias because of missing data	Bias in measurement of outcomes	Bias in selection of the reported result	
Colombo et al.^26^	Moderate	Moderate	Low	Low	Moderate	Low	Low	**Moderate**
Kessler et al.^25^	Moderate	Low	Low	Low	Low	Moderate	Low	**Moderate**
Basiri et al.^22^	Moderate	Low	Low	Low	Low	Low	Low	**Moderate**
Vilaseca et al.^23^	Moderate	Low	Low	Low	Low	Low	Low	**Moderate**
El‐Bahnasawy et al.^24^	Moderate	Moderate	Low	Low	Low	Low	Low	**Moderate**
Chen and Chiang^29^	Moderate	Moderate	Low	Low	Low	Low	Low	**Moderate**
Bai et al.^30^	Moderate	Low	Low	Low	Low	Low	Low	**Moderate**
Furrer et al.^31^	Moderate	Low	Low	Low	Low	Low	Low	**Moderate**
Gross et al.^32^	Moderate	Low	Low	Low	Low	Low	Low	**Moderate**
Bhatt et al.^34^	Serious	Moderate	Low	Low	Low	Low	Low	**Serious**
De Vries et al.^40^	Moderate	Low	Low	Low	Low	Low	Low	**Moderate**
Huang et al.^36^	Low	Moderate	Low	Low	Moderate	Moderate	Low	**Moderate**
Saad et al.^35^	Moderate	Moderate	Low	Low	Low	Low	Low	**Moderate**

ROBIN‐I = risk of bias in non‐randomized studies of interventions.

**TABLE 11:** Risk of bias assessment of case series studies using the Murad tool^19^

Study ID	Selection	Ascertainment		Causality				Reporting	Overall
	Do the patients represent the whole experience of the centre?	Was the exposure adequately ascertained?	Was the outcome adequately ascertained?	Were other alternative causes that may explain the observation ruled out?	Was there a challenge/recall phenomenon?	Was there a dose–response effect?	Was follow‐up long enough for outcomes?	Is there sufficient details to allow other investigators to replicate the research?	
Vallencien et al.^7^	Yes	Yes	Some concerns	Yes	N/A	N/A	Yes	Yes	**Some concerns**
Rozet et al.^27^	Yes	Yes	Yes	Yes	N/A	N/A	Yes	Yes	**Low risk**
Muto et al.^28^	Yes	Yes	Yes	Yes	N/A	N/A	Yes	Yes	**Low risk**
Voskuilen et al.^33^	Yes	Yes	Some concerns	Yes	N/A	N/A	Yes	Yes	**Some concerns**
Mertens et al.^39^	Yes	Yes	Yes	Yes	N/A	N/A	Yes	Yes	**Low risk**

### 3.6 Summary of findings

**TABLE 12:** Quality of evidence for each outcome as assessed by the GRADE system

Outcome	No. studies (no. participants)	Risk of bias	Imprecision	Inconsistency	Indirectness	Publication bias	Overall GRADE rating
Overall recurrence	4 (466)	Moderate	Moderate	Low	Low	Low	**Low**
Potency	6 (357)	Moderate	High	Low	Low	Low	**Very low**
Daytime continence	7 (753)	Moderate	Moderate	Low	Low	Low	**Low**
Night‐time continence	7 (753)	Moderate	Moderate	Low	Low	Low	**Low**

GRADE = Grading of Recommendations, Assessment, Development and Evaluations.

## 4 DISCUSSION

### 4.1 Summary of findings

Overall, 21 studies were included, comprising 3 RCTs, 14 cohort studies and 4 case series. The studies described prostate‐, capsule‐, seminal vesicle‐, nerve‐, uterus‐, ovary‐, vagina‐ and fallopian tube‐sparing techniques. Of these studies, only four involved women.

Thirteen studies analysed oncological outcomes, and no difference was found in overall recurrence in meta‐analysis (OR 0.70; 95% CI 0.44–1.10, *p* = 0.12). Fourteen studies reported on sexual function, including one female cohort study. The meta‐analysis for men showed significantly greater odds of potency preservation after OSC compared to radical (95% CI 5.07–16.16, *p* < 0.00001). Fourteen studies analysed urinary outcomes, including one female case series. In meta‐analysis for men, OSC was associated with significantly higher daytime (OR 2.61; 95% CI 1.74 to 3.92, *p* < 0.00001) and nighttime continence (OR 2.62; 95% CI 1.76 to 3.89, *p* < 0.00001) (Figures 4, 5, 6, 7).

### 4.2 Previous systematic reviews

Two previous systematic reviews comparing OSC with RC have been published, with both focussing on oncological and functional outcomes. One review compared the two techniques exclusively in men,^44^ and the other exclusively in women.^45^

The systematic review of male patients suggests that prostate‐, capsule‐, or nerve‐sparing cystectomy can lead to superior sexual outcomes without jeopardising oncological outcomes. This review also notes limitations, such as moderate quality evidence, and the need for carefully selecting patients for OSC.^44^ Selection criteria based on the age of the patient, type and position of bladder cancer, and family history of prostate cancer may influence the choice of prostate or capsule‐sparing approaches.

The systematic review of female patients concluded that organ‐sparing techniques were comparable to RC for oncological outcomes, with superior sexual and urinary function outcomes. However, this review comprised mostly small retrospective case series, with significant risk of bias and confounding.^45^ Our present review provides a more up‐to‐date and detailed analysis of organ‐sparing techniques in comparison with standard RC in both sexes. We include meta‐analyses of both oncological and functional outcomes, excluding smaller single‐arm studies and highlighting the need for more robust RCTs and female comparative studies.

### 4.3 Oncological outcomes

There is no consensus over the best surgical approach to balance oncological and functional outcomes. Indeed, RC is performed in patients with no observable evidence of disease in the surrounding organs.^46^ The disadvantage of RC in these patients is obvious: loss of sexual and urinary function is greatly associated with lower health‐related quality of life.^3^, ^5^ There is a preconceived notion that if RC is not performed, there will be a higher risk of local recurrence or metastatic disease, most likely conferring reduced mortality.^44^, ^45^

Interestingly, our meta‐analysis shows that there was no statistically significant difference in terms of overall recurrence between organ‐preserving and RC. However, this meta‐analysis is limited by the quality of evidence. Our findings suggest that OSC does not routinely need to be carried out to maintain similar oncological outcomes in carefully selected patients, which in turn could improve patients' quality of life. The patient selection should be based on age, oncological concerns, preoperative sexual function and postoperative expectations.^47^

When considering the studies that were not included in the meta‐analysis, there were relatively low rates of local recurrence and metastatic disease, which may suggest a similarity in overall recurrence between the two techniques. Ultimately, there is a paucity of reported data with only seven studies reporting on these oncological outcomes. As recurrence is an essential outcome for cancer surgery, further studies that assess for overall recurrence would help strengthen this analysis.

Further to the type of surgery, both patient age and preoperative cancer stage are important in dictating postoperative oncological outcomes. It was not possible to stratify postoperative oncological outcomes based on these two factors, and therefore, it is important to acknowledge that these confounding factors could contribute to the similar oncological outcomes reported.

Preoperative investigations, when accurate and reliable, should play an integral role in surgical planning ahead of both organ sparing and RC. Prostatic involvement, either by direct transmural infiltration or by urothelial spread arising in the prostatic urethra, is associated with worse oncological outcomes because of increased rates of urethral recurrence after cystectomy and worse mortality outcomes. Accurate identification of prostatic spread would be a key indicator in risk stratification for organ‐sparing surgery. The reported incidence of prostatic spread in patients with bladder cancer is between 16% and 48% in samples obtained by transurethral resection of the prostatic urethra and correctly identified in 89% of patients.^48^

When used in tangent with preoperative imaging methods such as magnetic resonance imaging (MRI), they may help guide bladder cancer risk stratification and the use of organ‐sparing techniques. Recent systematic review analysed the use of MRI as part of the Vesical Imaging Reporting and Data System (VI‐RADS), which can be used to distinguish MIBC from NMIBC using multiparametric MRI, concluding that its use confers good performance and reproducibility.^49^, ^50^ Though, its application in organ‐sparing risk stratification may be limited by its purported propensity to overstage bladder cancer in up to one third of patients.^51^

### 4.4 Functional outcomes

The rationale behind improved functional outcomes following OSC is that the sparing of nerves or various pelvic organs allows the preservation of neurovascular bundles that control potency and continence. Erectile function in males is dependent on parasympathetic innervation via the cavernous nerves, which travel to the penis via the pelvic and prostatic plexus and have a close anatomical relationship to the bladder, seminal vesicles, prostate and urethral sphincter.^52^ Similarly, the pelvic parasympathetic, lumbar sympathetic and pudendal nerves, which control urinary function, also lie in close proximity to these structures. RC involves complete excision of the bladder and surrounding structures, and this poses significant danger to these nerves.^53^ Our meta‐analyses show that OSC with neobladder formation provides favourable continence rates, daytime and nighttime potency rates when compared with standard RC.

Given both surgical options involve bladder reconstruction, it is important to analyse whether OSC has favourable postoperative urodynamics compared to standard RC. However, this was difficult in this review because only one study included postoperative urodynamic assessment results.

### 4.5 Limitations

OSC is an emerging technique in the treatment of bladder cancer. This systematic review provides an up‐to‐date analysis of this technique in both sexes from three important perspectives: oncological, sexual and urinary. However, this review does present with some limitations.

Firstly, the quality of the studies included were rated as either low or very low (Table 12). There were only three RCTs, and the majority of the studies are observational. Observational studies suffer from selection bias, and results often have to be used with caution.

Secondly, the reporting of outcomes between studies was inconsistent. In terms of oncological outcomes, there was incomplete reporting of overall survival, local and metastatic recurrence. Regarding functional outcomes, different methods were used to measure the same outcome, as evidenced through the use of both the IIEF‐5, which measured multiple domains associated with sexual function and the EHS, which relied solely on self‐reported erection strength.

There were inconsistencies with what each study defined as either continence or potency based upon the scoring questionnaires. For example, some papers classed patients as potent when able to achieve an erection with the use of medication such as alprostadil,^36^ whereas others defined potency as maintaining an erection without medication.^26^ Furthermore, not all studies specified the exact criteria for defining potency or continence.^9^, ^24^

The studies in this review showed a lack of standardised follow‐up duration. This may impact the present analysis of results because the data were recorded at different times. Furthermore, the lack of long‐term follow‐up limits the use of the meta‐analysis in predicting any lasting effects of OSC.

There is a significant lack of female studies. This is likely because of the greater prevalence of bladder cancer amongst males when compared with females.^54^ However, when looking at the outcomes, there is research showing females actually have a greater risk of readmission after cystectomy than males because of complications.^55^ Although a previous systematic review included 15 studies analysing OSC in females, these studies did not meet the inclusion criteria because of small sample sizes or being single‐arm studies.^56^

This review has also not discussed further complications of the surgery, for instance, the rate of bleeding, infection, bowel obstruction and urethral strictures. Analysing the rates of these complications, in the context of OSC and RC, can further provide a more detailed comparison of these two techniques, especially given the morbid nature of cystectomy.

### 4.6 Recommendations

1. Based on the low quality evidence in this review, there is potential for OSC to be considered where possible, instead of RC, when deciding the surgical management of carefully selected bladder cancer patients in order to preserve sexual and urinary function. Surgical planning may be aided by the use of preoperative imaging tools such as VI‐RADS.
2. Future OSC RCTs, especially comparing oncological outcomes with standardised outcome reporting and long‐term follow‐ups, as well as female studies, are needed to add to the evidence base.
3. Research comparing single versus multi‐organ sparing cystectomy and comparing different types of OSC will be beneficial.

## 5 CONCLUSION

There is a potential advantage to OSC regarding sexual and urinary function with equivalent oncological outcomes in carefully selected men when compared to RC. Our results would benefit from more standardised functional outcome reporting, further study of oncological outcomes in robust RCTs and higher quality OSC studies in women.

## ACKNOWLEDGEMENT

This work was supported and funded by King's College London [JISC].

## CONFLICTS OF INTEREST

All authors declare no conflicts of interest as per the ICMJE COI.

## AUTHOR CONTRIBUTIONS

All Authors contributed to the above review.
